# Neural correlates of anxiety under interrogation in guilt or innocence contexts

**DOI:** 10.1371/journal.pone.0230837

**Published:** 2020-04-09

**Authors:** Sole Yoo, Hanseul H. Choi, Hae-Yoon Choi, Sungjae Yun, Haeil Park, Hyunseok Bahng, Hyunki Hong, Heesong Kim, Hae-Jeong Park

**Affiliations:** 1 Department of Cognitive Science, Yonsei University, Seoul, Republic of Korea; 2 Department of Nuclear Medicine, Department of Psychiatry, Severance Hospital, Yonsei University College of Medicine, Seoul, Republic of Korea; 3 Center for Systems and Translational Brain Sciences, Institute of Human Complexity and Systems Science, Yonsei University, Seoul, Republic of Korea; 4 BK21 PLUS Project for Medical Science, Yonsei University College of Medicine, Seoul, Republic of Korea; 5 Department of English Literature, Kyung Hee University, Seoul, Republic of Korea; 6 The National Forensic Service, Wonju-si, Gangwon-do, Republic of Korea; Universita degli Studi di Udine, ITALY

## Abstract

Interrogation elicits anxiety in individuals under scrutiny regardless of their innocence, and thus, anxious responses to interrogation should be differentiated from deceptive behavior in practical lie detection settings. Despite its importance, not many empirical studies have yet been done to separate the effects of interrogation from the acts of lying or guilt state. The present fMRI study attempted to identify neural substrates of anxious responses under interrogation in either innocent or guilt contexts by developing a modified “Doubt” game. Participants in the guilt condition showed higher brain activations in the right central-executive network and bilateral basal ganglia. Regardless of the person’s innocence, we observed higher activation of the salience, theory of mind and sensory-motor networks–areas associated with anxiety-related responses in the interrogative condition, compared to the waived conditions. We further explored two different types of anxious responses under interrogation–true detection anxiety in the guilty (true positive) and false detection anxiety in the innocent (false positive). Differential neural responses across these two conditions were captured at the caudate, thalamus, ventral anterior cingulate and ventromedial prefrontal cortex. We conclude that anxiety is a common neural response to interrogation, regardless of an individual’s innocence, and that there are detectable differences in neural responses for true positive and false positive anxious responses under interrogation. The results of our study highlight a need to isolate complex cognitive processes involved in the deceptive acts from the emotional and regulatory responses to interrogation in lie detection schemes.

## Introduction

When people are being interrogated, they naturally exhibit signs of nervousness and somatic tensions elicited by the sympathetic nervous system [1–3]. These sympathetic responses can occur due to the anxiety caused by merely expecting imminent threat or by over-reacting to it. Interrogation also elicits various cognitive responses, such as memory retrieval, internal regulation of anxiety, theory of mind (i.e., trying to understand the interrogator’s perspective), and suppression of guilty conscience (or remorse) [4, 5]. With this understanding of the heterogeneous nature of elements involved in the anxious response to interrogation, it should be noted that anxious response under interrogation is not only caused by the actual deception (we call true positive anxiety) in a guilt situation but also caused by the fear of being falsely accused (false positive anxiety) in an innocent situation [6, 7]. This distinction stems not from the emotional or psychophysical manifestation of anxiety, but from different causes of anxiety. This point is of special importance particularly when lie detection strategies solely rely on the emotional arousal indicated by physiological responses elicited via the sympathetic system.

In search of a more accurate lie detection system, researchers, over the last two decades, have increasingly investigated neural responses observed by functional MRI (magnetic resonance imaging). Despite the heterogeneity in their designs, most of these studies fundamentally focused on identifying neural substrates underlying deception and truth-telling while participants performed a relevant task. For example, one study used a modified version of the Guilty Knowledge Test (GKT) [8] and asked participants to lie about their possession of a target stimulus (a specific kind of playing card) while answering truthfully to all other cards [9]. Another study evaluated the patterns of neural activity among different types of lies (e.g. spontaneous-isolated and memorized-scenario lies)[10], and yet another study used a modified GKT paradigm in which the participants were asked to lie about their possession of cards according to given instructions [11]. Yang and colleagues[12] examined brain regions capable of decoding true-thoughts, after making the participants tell a lie with an instructed cue.

Despite a sizeable volume of research about the neural correlates of deception, to our knowledge, no empirical study has yet been done to examine the effects of situational pressure of interrogation on neural responses related to anxiety and other accompanying behaviors (e.g., trying to hide one’s own biophysical responses) following either a deceptive or truthful act. We believe this topic should be an important line of research on deception because an act of lying frequently takes place in an interrogative context. After all, a stressful interrogation setting has such potent anxiogenic effects on the subject that it has often led to innocent people being wrongfully convicted [13, 14]. Therefore, in a practical setting of lie detection, it is critical to isolate the complex emotional and regulatory responses under interrogation from the internal processes following the deceptive act itself.

For this purpose, we designed an event-related fMRI task, in which we attempted to differentiate the anxious response for interrogation from responses for being in guilt situation. We incorporated a card game called “Doubt”, which involves the participant’s possession of target playing cards as a basis for decision to deceive with the knowledge of an impending interrogation about that decision. After telling a lie or the truth during the game, participants were either implicitly interrogated (based on facial expression or biophysical signals) or the interrogation was waived off. We then analyzed brain responses related to a sense of anxiety during the interrogation or a sense of relief during the waiting period in both the guilt and innocent conditions.

As we have previously discussed, interrogation elicits a complex combination of neural responses comprising both affective and cognitive processes related to anxiety–the obvious anxious responses as reflected in the sympathetic responses and the cognitive processes recruited to regulate one’s own anxiety. The first is rather a passive, negative, emotional response to the interrogation that unfolds almost automatically while the second is a more active response to the interrogation that include processes such as mentalizing about the interrogator’s view of the participant and changing one’s own facial expressions to appear more innocent. Therefore, we speculated that two major brain networks would be involved in these processes: the salience network and a network responsible for theory of mind or mentalization. In the case of waive (no interrogation) condition, we expected that people would feel more relaxed, which would result in the activation of the default mode network (DMN) [15–17].

These regions involved in the interrogation process may differ from regions observed in deceptive acts investigated by previous studies mentioned above. Previous literature have identified a variety of brain regions as neural substrates of deception: 1) areas in the frontal cortex, such as the ventrolateral prefrontal cortex, dorsolateral prefrontal cortex, dorso-medial prefrontal cortex, anterior cingulate cortex, orbitofrontal cortex, and superior frontal gyrus; 2) regions in the temporal cortex, such as the superior temporal sulcus and para-hippocampal gyrus; and 3) other regions such as the precuneus, supramarginal gyrus, caudate, and thalamic nuclei [4, 9–12, 18–20].

By analyzing main effects, we expected to show that anxious responses have a sort of commonality for interrogation regardless of one’s guilty or innocent state. We also expected to find differential neural substrates of true-positive and false-positive anxiety in the interaction contrast. This study highlights a need for isolating the complex emotional and regulatory responses under interrogation from the processes involved in the guilty or innocent act itself.

## Methods

### Participants

Twenty-two young, healthy participants with no history of neurological or psychiatric impairment participated in this study. Data from three participants were excluded due to poor task performance and excessive motion in the scanner, allowing data from 19 participants in total (11 males, 19–31 years old, mean age 25.68 (standard deviation, s.d. = 3.58)) to be included in the final analysis. Informed consent was obtained from each participant prior to the experiment, in accordance with a protocol sanctioned by Severance Institutional Review Board.

### Task design

We incorporated interrogation into an experimental design to elicit anxiety-related responses from the participants. Interrogators (confederates) were introduced to the participants as professional interrogators to elicit a sufficient sense of anxiety. The participants were told that the facial expressions and biophysical signals gathered from the devices (video camera, electrocardiogram, electromyogram and respiration sensors) attached to them will be used to determine their truthfulness, although a computerized program was instead making the interrogation judgment pseudo-randomly without using any biophysical information from the participant (at a rate of 60% and 80%). To evoke a lie, monetary rewards were utilized as the potential lie-eliciting incentive and forfeit of these rewards as a form of penalty, while keeping in accordance with the ethical standards that an experiment must not provoke too serious a moral dilemma for the participants. A modified form of a card game was introduced and the trial was repeated for a sufficient number of times, given the fact that fMRI experiments require multiple trials of data for there to be any meaningful signal detection.

The card game used as the main cognitive task is a modified version of a game called “Doubt”, in which players must try to get rid of all the cards that were distributed to each of them at the beginning of the game; a player who ends up discarding the given cards first is deemed the winner.

The “Doubt” paradigm was revised to fit the current fMRI experiment as follows: (1) The trials are played in a set. In each set, participants are randomly given four cards to discard: three that have a number ranging from 2 to 7 and one Pass card. In every trial, a number from 2 to 7 is randomly presented on the screen, and if the participant happens to have a card with the same number, s/he can discard it, but if s/he doesn’t, s/he can either use the Pass card to skip to the next trial or deceptively discard an incompatible card. Participants are told that they would receive a basic prize of 5000 won (equivalent of 5 USD) if they manage to discard all three number cards in four trials (with the use of the Pass card). The participants are also told that they would receive 10000 won (basic prize of 5000 won + bonus of 5000 won) if they manage to discard all three number cards in three trials (without the use of the Pass card and without being detected as deceptive). They are told that if the deception is ever discovered by the interrogator for a given set, they would lose 5000 won as penalty (-5000). They are supposed to discard all the cards at most in four trials in each trial set (three trials without the use of the Pass card), forcing them to lie on some occasions even though they may not want to. A round of these three or four trials needed to discard the three given (number) cards makes up a set. (2) After several repeated sets like this (the way we determined the number of repetitions of these sets for each participant is explained later), the accumulated prize money shown after the final trial counts proportionally as the final compensation.

For the two seconds after the participants make the decision (discarding a card by pressing a button), a sentence “The dealer is watching you” is displayed on the screen while the participants wait for the decision about whether they will be placed under interrogation. Every trial is followed by either an interrogation to determine whether s/he has discarded a card truthfully or not, or a simple waiting period without any interrogation (no interrogation condition); whether or not there will be an interrogation is determined by the computer program designed by the researchers in such a way that there are at least 10 trials of each of the four conditions (guilt & interrogation, innocence & interrogation, guilt & no-interrogation, innocence & no-interrogation) for each participant.

During the interrogation, a sentence “Did you truly discard n (the number presented)?” is presented on the screen. During the waiting period (no-interrogation condition), participants see “Please stay until the next trial” on the screen. The participants do not have to answer the interrogation question with a yes or no, but the question is presented to the participants simply to imply that the interrogators are determining their truthfulness by looking at their facial expressions and biophysical signals as they have been told. The interrogation or waiting period lasts for seven seconds, after which a fixation cross is displayed for three seconds until the next trial. When the participant discards the final number card (the third card), the amounts of current and accumulated money are presented for two seconds. After this a fixation cross is displayed for three seconds until the next trial.

**Fig 1:**
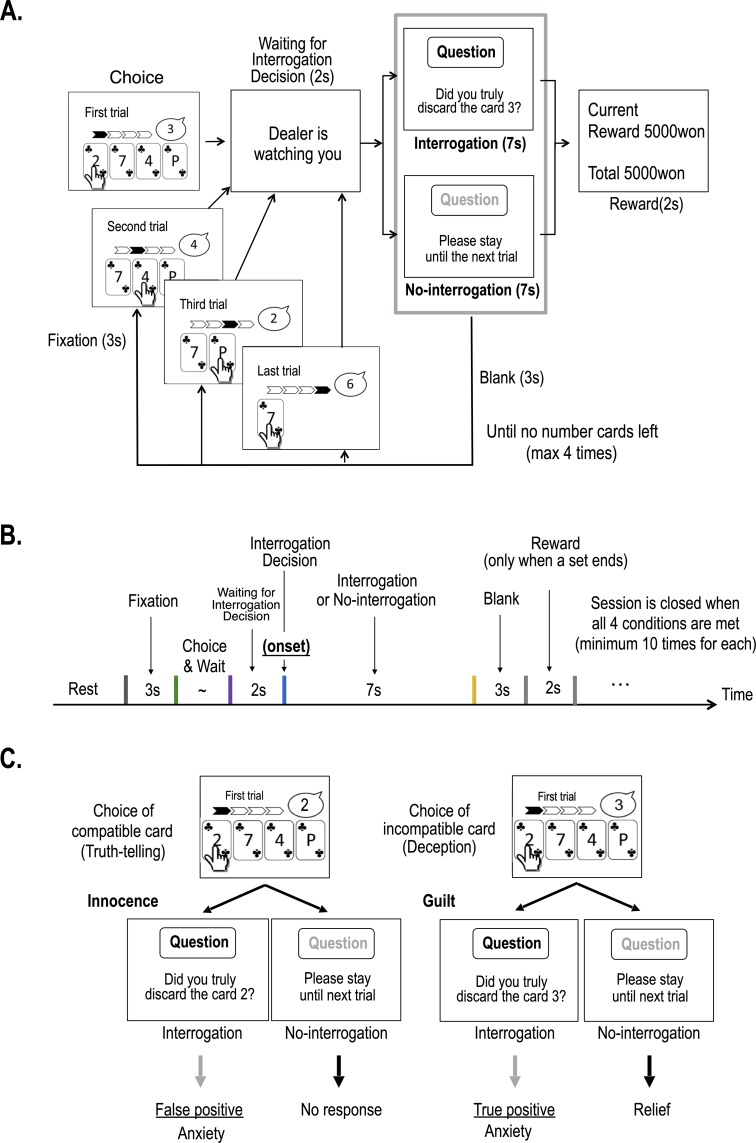
The fMRI experiment paradigm. (A) Experimental procedure for the modified version of “Doubt” game under interrogation used in the current study (B) time scheme for the procedure. (C) Description of two different types of anxiety, i.e., true positive and false positive anxiety under guilt and innocence conditions, respectively.

Note that, since the computer program generated the target stimulus numbers and received inputs from each participant, the program was aware of the truthfulness of each decision made by the participant without analyzing any biophysical signals, and made a pseudo-random judgement, meeting a predetermined judgement accuracy (60% for the first session). We assumed that the participants might reckon how accurate the judgement is by looking at the monetary rewards presented after finishing each set (three or four trials). However, the procedures were carefully managed to make the participants believe that it was a human interrogator, not a computer program behind the device, and we assumed this would motivate the participants to do their best to manage their facial and biophysical signals to deceive a human interrogator in their efforts to acquire the biggest final compensation possible. The experimental procedures are summarized in Fig 1.

The task was conducted in two sessions. All the aspects of the procedures in both sessions were the same except for the participants’ sense of the interrogator’s judgment accuracy. We implied to the participants that the judgment accuracy rate in the second session would be higher than that in the first session by telling them that the interrogator will use individuals’ information from the first session to judge their truthfulness in the second session. The interrogation judgment accuracy of the computer program was simply enhanced to an 80% in the second session from a 60% in the first session.

After the study, the participants were debriefed that it was a computer program that was judging their truthfulness. The participants were given the final compensation (roughly proportional to the prize money accumulated) within a range of minimum 50000 to maximum 80000 won (approximately 50–80 USD).

### Procedure

The participants were given a thorough orientation on the cognitive tasks involved in the experiment while meeting with a confederate introduced as a professional interrogator. The participants were told that this interrogator will discern their truthfulness by observing their facial expressions and measuring their biophysical signals (pupil sizes, heart rates, breathing, and electromyograms) during fMRI scanning. The polygraph equipment (clinical MR-compatible EKG and respiratory monitoring) was not operating, but simply there to amplify the participants’ anxiety. All the participants agreed to these conditions and provided written informed consent.

The participants performed the tasks in two successive fMRI sessions, with the polygraph instruments attached to their bodies. The cognitive tasks were delivered through Psychtoolbox 3 (http://psychtoolbox.org/).

There are 4 possible conditions in which the participants could proceed with each trial: 1) innocence & interrogation condition (in which the participant goes through interrogation after truthfully discarding a compatible card), 2) innocence & no-interrogation condition (in which the participant goes through a simple waiting period after truthfully discarding a compatible card), 3) guilt & interrogation condition (in which the participant goes through interrogation after deceptively discarding an incompatible card, and 4) guilt & no-interrogation condition (in which the participant goes through a waiting period after deceptively discarding an incompatible card). We programmed an algorithm in such a way that the trials would run repeatedly so that each of these four conditions were recorded at least 10 times for each participant in a pseudo-random but adaptively administered manner.

After these procedures were over, the participants were asked to fill out a post-hoc questionnaire allowing them to describe how they felt about having gone through each of the four conditions, the two successive sessions, and the overall experience of the experiment. In addition, they were asked to fill out an anxiety assessment questionnaire in which they were to rate the level of anxiety experienced during each of the 4 conditions [from -5 (very relaxed) to +5 (very anxious)].

### Data acquisition, processing, and statistical analysis

Brain activity was measured using a Siemens 3T MRI system (Siemens MAGNETOM Trio, Germany) with T2* weighted single shot echo planar imaging (EPI). For each task, fMRI images with four dummy scans were acquired axially with the following parameters: repetition time (TR) = 2000ms, echo time (TE) = 20ms, flip angle = 90°, number of slices = 42, interleaved sequence, 3mm slices with no gap, FOV = 22cm, matrix size = 80x80.

Head movement was minimized by adding foam pads into the head coil. To facilitate later spatial normalization, we also obtained a high-resolution T1-weighted MRI volume dataset for each subject with a 3D T1-TFE sequence configured with the following acquisition parameters: axial acquisition with a 224 × 224 matrix, 220 mm field of view, 0.9 × 0.9 × 1.0 mm voxel unit, 2.6 ms TE, 1900 ms TR, 9° flip angle, and 0 mm slice gap.

Image preprocessing was carried out by using statistical parametric mapping (SPM12, http://fil.ion.ucl.ac.uk/spm, Wellcome Department of Cognitive Neurology, London, UK) [21]. The procedure included slice timing correction for the interleaved sequence, motion correction by realigning all the images to the first fMRI image, normalization to standard Montreal Neurological Institute (MNI) template in SPM12, and smoothing with a 6-mm full-width-at-half-maximum (FWHM) Gaussian filter. Low-frequency drifts were removed using a high-pass filter with a cut-off frequency of 128 seconds. In the individual level analysis, the six motion regressors that were calculated during realignment procedure were added to eliminate unnecessary effect caused by head movement.

The moment of interrogation notification was used as an onset time in the generalized linear model analysis of the first level. fMRI signals at every voxel were modeled with regressors, one for each condition comprising every event for the condition with a duration of 7 sec after the onset time, convolved with the canonical hemodynamic response function.

Group-level activation was evaluated using a random effect model for the effect of interest. Statistical difference in activation among conditions was estimated using a flexible design with two factors: 1) a situation following discarding compatible cards (“innocence” condition) versus a situation following discarding incompatible cards (“guilt” condition); and 2) interrogation versus no interrogation. To minimize the effects of the differences in the number of trials for each condition, we assigned the number of trials as a nuisance variable in the group level analysis.

For the group level inference of SPM results, statistical significance was defined by the clusters surviving the voxel-level threshold of p < 0.001 (uncorrected) and the cluster-level extent threshold of p < 0.05 (cluster size ≥ 112 voxels for FWHM 10.2 mm) generated with 10,000 Monte Carlo simulations using the 3dClustSim program (September 2017 version) in the Analysis of Functional NeuroImages software (AFNI; https://afni.nimh.nih.gov/afni).

As a post-hoc analysis, the percent signal changes for significantly detected clusters were calculated by counting 3 mm-diameter sphere regions around the peak of the cluster using MarsBaR software [22]. The percent signal change measures were taken to explore the directions of interaction between the two factors; Guilt vs. Innocence and Interrogation vs. Non-interrogation, rather than to derive novel inferences from them.

## Results

### Behavioral data

Two-way repeated measures analysis of variance (ANOVA) for the response times of guilt and innocence conditions (except for one missing data) shows a significant difference between guilt (mean ± s.d. in sec: 3.44 ± 1.17) and innocence (3.10 ± 0.45) conditions (*F* (1, 71) = 8.349, *p* = 0.005, partial eta-squared ηp2 = 0.19).

Two-way repeated measures ANOVA for post-hoc anxiety scores (self-reports) shows a significant main effect for anxiety scores in the contrast between interrogation (1.05 ± 0.57) versus no-interrogation (-3.13 ± 0.40) conditions (*F* (1, 18) = 39.757, *p* = 0.000, ηp2 = 0.52). There was also a significant difference between the anxiety score of guilt (mean ± s.d.: -0.63 ± 0.32) versus innocence (-1.45 ± 0.48) conditions (*F* (1,18) = 4.68, *p* = 0.044, ηp2 = 0.12). Interaction between the two factors was significant (*F* (1,18) = 19.49, *p* = 0.000). Paired *t*-test as a post-hoc showed that the guilt & interrogation condition (mean ± s.d.: 2.05 ± 2.41) exhibited higher anxiety scores than the innocence & interrogation condition (0.05 ± 2.93) (*t*_18_ = 4.14, p = 0.001, Cohen’s d indicating effect size *d* = 0.75). The innocence & interrogation condition yielded a higher score of anxiety than both the guilt & no-interrogation (-3.32 ± 1.95) (*t*_18_ = 3.97, *p* = 0.001, *d* = 1.35) and innocence & no-interrogation conditions (-2.95 ± 2.04) (*t*_18_ = 4.59, *p* = 0.000, *d* = 1.19).

13 out of 19 participants reported that they were anxious during interrogation even when they were telling the truth, and 9 participants reported having attempted to engage in autosuggestion or control their biophysical signals to deceive the interrogator. These reports were given voluntarily with a prompt asking participants to describe what they felt during the experiment rather than directing the participants to answer specifically to a yes or no question. Therefore, more participants may have felt anxious and engaged in manipulation of their biophysical signals yet decided not to include these details in their description.

As for the session, only half of all participants (11) reported that the judgment accuracy had seemed to be higher in the second sessions. In addition to this high inter-individual difference in the sense of judgement accuracy for the second session, we could not rule out compounds from the fixed-order session manipulation (lower to higher accuracy). Therefore, we did not consider to analyze the session effects independently.

Participants conducted on average 17.6 (s.d. 2.4) sets (three or four trials per a set) for approximately 30 minutes. The number of trials executed for each condition type for the two sessions was as follows: guilt & interrogation (mean ± s.d.: 24.44 ± 4.44), guilt & no-interrogation (28.56 ± 8.10), innocence & interrogation (25.89 ± 5.70) and innocence & no-interrogation (26.44 ± 5.90). Two-way repeated measures ANOVA of the mean numbers of executed trials for each condition type showed no significant effect for guilt (26.50 ± 1.39) versus innocence (26.17 ± 1.20) (*p* = 0.886, *d* = 0.001) but significant for interrogation (25.17 ± 0.51) versus non-interrogation (27.50 ± 1.01) (*p* = 0.035). Participants who deceived more performed more trials (r = 0.59, p = 0.01), and received more rewards (r = 0.6, p = 0.009). However, interrogation decision was not dependent on deceptive trials (r = 0.14) since it was determined by computer semi-randomly within the preset accuracy.

### fMRI imaging data

**Fig 2:**
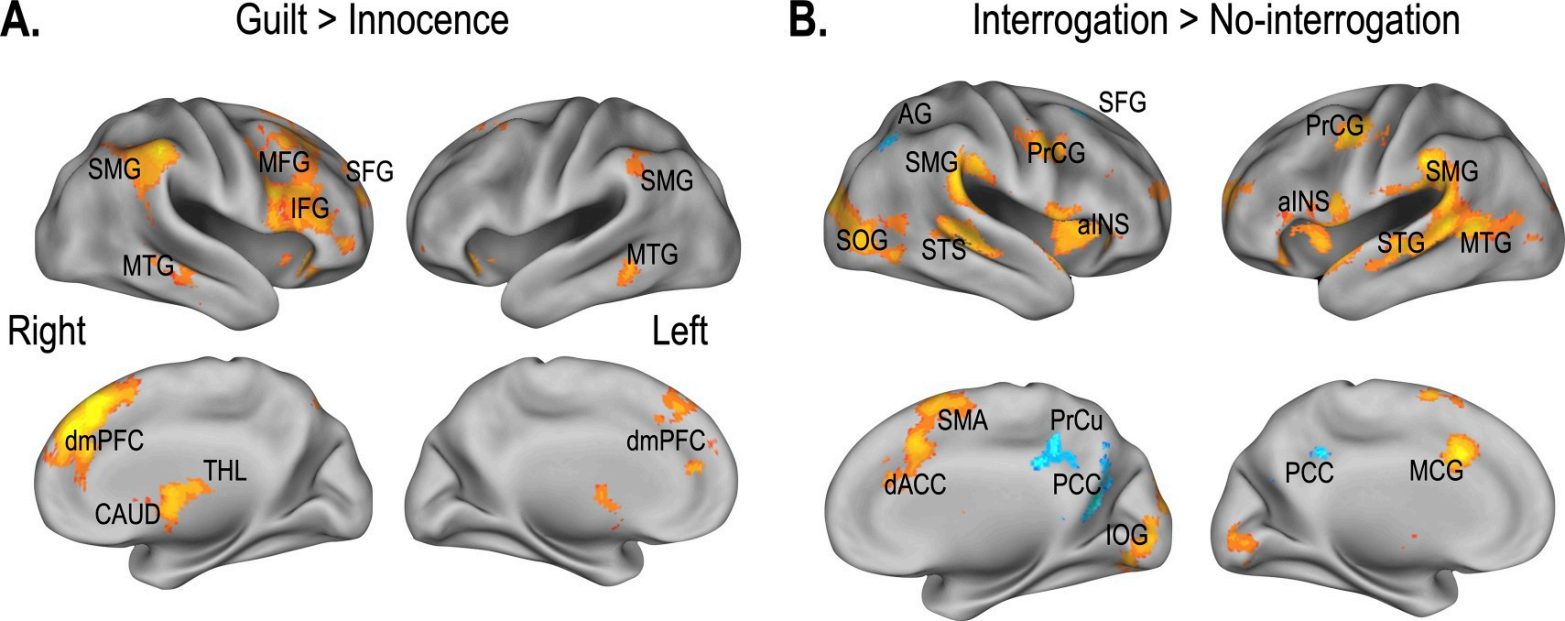
Statistical parametric maps for main effects. (A) Brain regions detected to be involved in the Guilt versus Innocence contrast are shown. (B) Brain regions detected to be involved in the Interrogation versus No-interrogation contrast are shown (red: increased activation, blue: decreased activation). IFG: inferior frontal gyrus, SFG: superior frontal gyrus, MFG: middle frontal gyrus, MTG: middle temporal gyrus, SMG: supramarginal gyrus, dmPFC: dorsal medial prefrontal cortex, CAUD: caudate, THL: thalamus, STG: superior temporal gyrus, STS: superior temporal sulcus, SOG: superior occipital gyrus, PrCG: precentral gyrus, aINS: anterior insular, IOG: inferior occipital gyrus, SMA: anterior supplementary motor area, dACC: dorsal anterior cingulate cortex, MCG: middle cingulate gyrus, AG: angular gyrus, SFG: superior frontal gyrus, PCC: posterior cingulate cortex, PrCU: precuneus.

Brain activation results from fMRI data concerning the main effects of guilt vs. innocence and of interrogation vs. no interrogation are displayed in Fig 2 and Table 1.

**Table 1:** Brain activation corresponding to the effects of Interrogation, Guilt and interactions.

Region	Coordinate (x,y,z)	Zmax	Cluster Size	Region	Coordinate (x,y,z)	Zmax	Cluster Size
*Interrogation> No-interrogation*				*Interrogation < No-interrogation*			
L Superior Temporal Gyrus (BA 22)	-62,-42,18	6.23	2930	R Precuneus (BA 7)	12,-56,20	5.19	885
L Supramarginal Gyrus (BA 40)	-60,-36,34	5.89	-	R Posterior Cingulate Cortex (BA 23)	2,-38,34	4.25	-
L Middle Temporal Gyrus (BA 21)	-52,-44,8	5.70	-	R Angular Gyrus (BA 39)	42,-72,44	4.76	374
L Middle Cingulate Gyrus	-10,12,34	6.20	2311	R Middle Frontal Gyrus (BA 9)	30,14,54	4.58	173
R Supplementary Motor Cortex (BA 6)	6,16,40	5.63	-	R Superior Frontal Gyrus (BA 8)	30,20,60	4.09	-
L Supplementary Motor Cortex (BA 6)	-6,2,66	5.30	-	L Posterior Cingulate Cortex (BA 31)	-12,-46,34	4.32	202
R dorsal Anterior Cingulate Cortex (BA 32)	8, 26,26	4.66		L Precuneus (BA 7)	-16,-56,22	3.76	-
R Temporal Pole (BA 38)	50,14,-8	6.12	2499				
R Anterior Insula (BA 13)	44,8,-2	5.82	-				
L Temporal Pole (BA 38)	-50,14,-10	6.09	2799	*Guilt > Innocence*			
L Caudate	-14,0,8	5.57	-	R Medial Superior Frontal Gyrus (BA 8)	2,32,50	6.70	10462
L Anterior Insula (BA 13)	-42,8,0	5.43	-	R Superior Frontal Gyrus (BA 8)	6,48,46	5.66	-
R Supramarginal Gyrus (BA 40)	64,-38,30	5.81	5091	R Inferior Frontal Gyrus (BA 44)	54,28,18	5.38	-
R Inferior Occipital Gyrus (BA 19)	44,-82,0	5.73	-	R Anterior Insula (BA 13)	30,24,-4	6.18	-
L Precentral Gyrus (BA 4)	-46,-2,42	5.75	785	R Angular Gyrus (BA 39)	52,-50,54	6.04	2775
L Middle Frontal Gyrus (BA 6)	-48,4,50	5.37	-	R Supramarginal Gyrus (BA 40)	50,-40,44	6.03	-
R Precentral Gyrus (BA 4)	50,4,50	5.62	744	R Middle Temporal Gyrus (BA 21)	58,-28,-8	4.16	-
R Middle Frontal Gyrus (BA 46)	38,2,36	4.59	-	R Thalamus	2,-10,12	5.10	859
L Middle Frontal Gyrus (BA 10)	-28,50,18	4.86	632	R Caudate	14,8,12	4.19	-
L Cerebellum	-30,-64,-26	4.64	112	L Thalamus	-8,-6,8	3.94	-
L Superior Occipital Gyrus (BA 19)	-16,-88,40	4.28	219	L Anterior Insula (BA 13)	-32,20,-6	5.01	226
L Middle Occipital Gyrus (BA 19)	-34,-92,14	3.86	-	L Cerebellum	-14,-78,-34	4.88	368
R Superior Frontal Gyrus (BA 8)	26,56,20	4.23	344	L Angular Gyrus (BA 39)	-48,-54,54	4.49	589
R Middle Frontal Gyrus (BA 9)	34,54,26	3.73	-	L Supramarginal Gyrus (BA 40)	-60,-48,42	4.25	-
				L Middle Temporal Gyrus (BA 21)	-58,-46,-10	4.47	202
*Guilt < Innocence*				L Lateral Orbital Gyrus (BA 11)	-44,52,-12	3.90	130
Not detected				L Middle Frontal Gyrus (BA 46)	-50,46,0	3.61	-
				R Precuneus (BA 7)	14,-68,40	3.74	126
*(Guilt–Innocence) × (Interrogation–No-interrogation)*				*(Innocence- Guilt) × (Interrogation–No-interrogation)*			
R Caudate	12,6,10	4.33	443	Not detected			
R Thalamus	4,0,12	4.20	-				
L Thalamus	-10,0,6	4.08	-				
L Cerebellum	-20,-42,-26	3.75	149				
L Ventromedial Prefrontal Cortex (BA 10)	-2,52,-6	3.71	112				
R Ventromedial Prefrontal Cortex (BA 11)	4,44,-12	3.37	-				
L Anterior Cingulate Cortex (BA 32)	-2,40,4	3.67	101*				
R Anterior Cingulate Cortex (BA 32)	8,38,12	3.54	-				

*p* < 0.001, cluster size > 112

* not significant in terms of cluster size criteria > 112 (corrected p < 0.05) but showing a tendency of significance. BA = Brodmann Area; L = Left; R = Right; Coordinate = Montreal Neurological Institute (x,y,z); Zmax = Z maximum within a cluster. “-” in the cluster size indicates that this coordinate is a peak location that belongs to the cluster listed immediately above.

Guilt conditions, compared to innocence conditions, yielded increased activation mainly in the right medial superior frontal gyrus, right superior frontal gyrus, right inferior frontal gyrus, bilateral anterior insula, bilateral angular gyrus, bilateral thalamus, bilateral supramarginal gyrus, bilateral middle temporal gyrus, right caudate, left cerebellum, left lateral orbital gyrus, left middle frontal gyrus and right precuneus. The increased activation was more dominant in the right hemisphere than the left (Fig 2A).

Relative to no-interrogation conditions, interrogation conditions elicited significantly higher activation in the left superior temporal gyrus, bilateral supramarginal gyrus, left middle temporal/cingulate gyrus, bilateral supplementary motor cortex, right dorsal anterior cingulate cortex, bilateral temporal pole, bilateral precentral gyrus, bilateral middle frontal gyrus, bilateral anterior insula, left caudate, right inferior occipital gyrus, left superior/middle occipital gyrus, left cerebellum, right superior frontal gyrus and bilateral middle frontal gyrus. No-interrogation conditions, relative to interrogation conditions, yielded higher activation in the bilateral posterior cingulate, bilateral precuneus, right angular gyrus, and right superior/middle frontal gyrus (Fig 2B).

**Fig 3:**
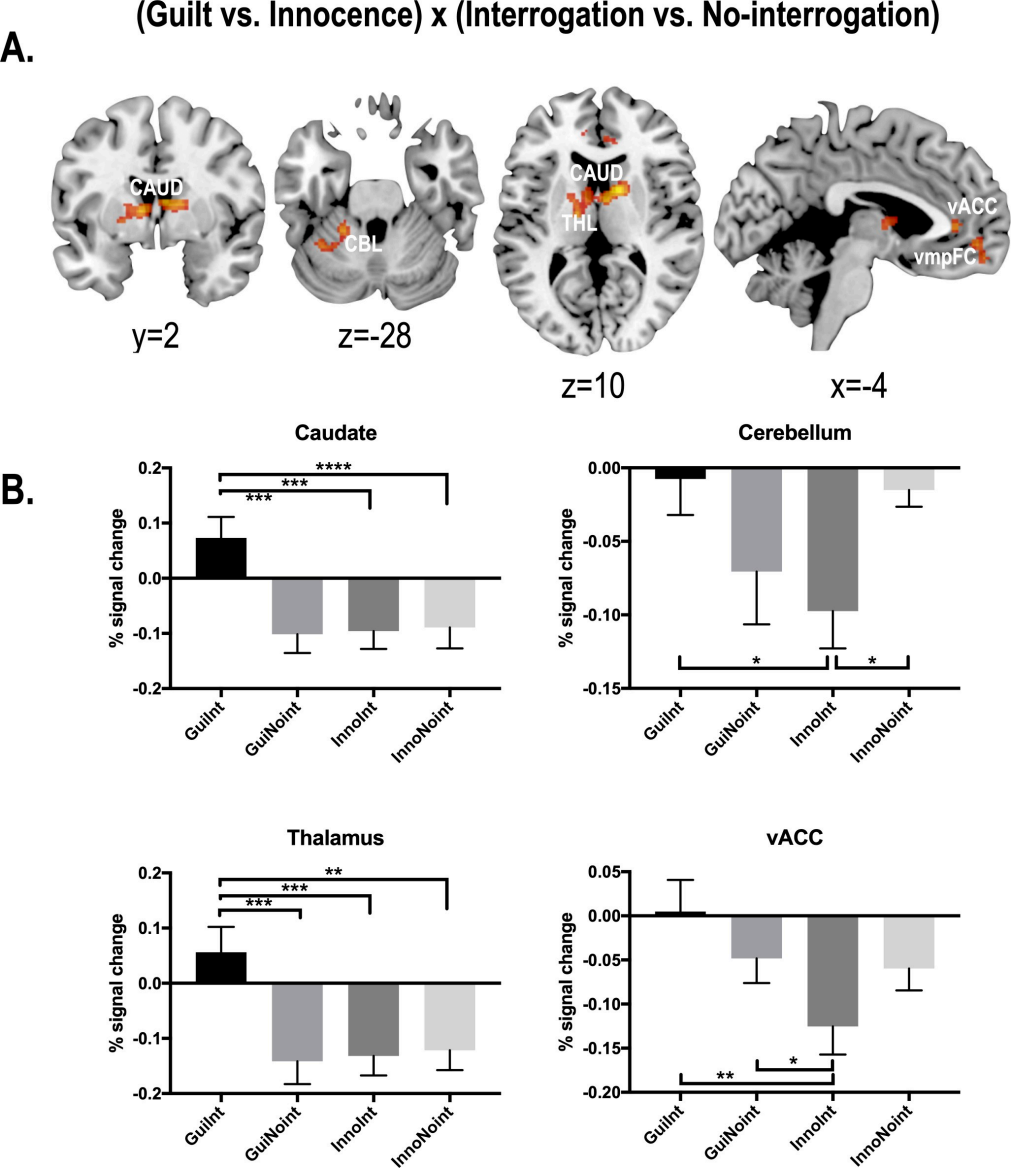
Statistical parametric maps for interaction between guilt vs. innocence and interrogation vs. no-interrogation. (A) Statistical map for interaction; (Guilt—Innocence) x (Interrogation—No-interrogation) displayed over slices. (B) Percent signal changes (mean and standard error) are displayed for GuiInt (guilt & interrogation), GuiNoint (guilt & no-interrogation), InnoInt (innocence & interrogation) and InnoNoint (innocence & no-interrogation) at the right caudate (x/y/z = 12/6/10), cerebellum (-20/-42/-26), left thalamus (-10/0/6) and left ventral anterior cingulate cortex (-2/40/4). *, ** and *** indicate p < 0.05, p < 0.01 and p < 0.001, respectively. CAUD: caudate, THL: thalamus, CBL: cerebellum, vACC: ventral anterior cingulate cortex, vmpFC: ventromedial prefrontal cortex. x,y, z indicates MNI coordinates in mm.

Interactions between guilt vs. innocence and interrogation vs. no-interrogation were found at the bilateral thalamus, right caudate, left cerebellum, bilateral ventromedial frontal cortex and ventral anterior cingulate cortex (Table 1). Fig 3 displays brain regions of interaction effects and their percent signal changes to show the directions of the interactions at four major regions (the caudate, cerebellum, thalamus and ventral anterior cingulate cortex). The percent signal changes showed increase (or non-negative) only in the guilt and interrogation condition while all the other conditions exhibited a suppressed direction.

For reference, here we present statistical analysis results to show the size of interactive effects at the four regions. Repeated measures ANOVA for the percent signal changes at the thalamus and the caudate revealed a significant difference between guilt & interrogation conditions and guilt & no-interrogation conditions (p = 0.0008 for the thalamus and p = 0.001 for the caudate, adjusted by Sidak’s multiple comparison test), between guilt & interrogation conditions and innocent & interrogation conditions (adjusted p = 0.001 and p = 0.0004), and between guilt & interrogation conditions and innocence & no-interrogation conditions (adjusted p = 0.0018 and p < 0.0001). The cerebellum showed a significant difference between guilt & interrogation and innocence & interrogation (adjusted p = 0.0242) and between innocence & interrogation and innocence & no-interrogation (adjusted p = 0.0205). The ventral anterior cingulate gyrus showed statistical significance only in the contrast between guilt & interrogation and guilt & no-interrogation (adjusted p = 0.0365). Since the regions of interests were chosen from the results of the current study, the statistical analysis results for the four regions were provided just to elaborate on the results, rather than to produce a novel finding.

## Discussion

Previous studies on the neural processes of lying and lie detection have not separated anxious responses under interrogation from the deceptive act or its post-hoc responses, which occur in tandem in practical lie detection settings. In the current study, we neurobiologically tested the common knowledge that all interrogations elicit anxiety (as well as cognitive processes to hide anxiety) regardless of the actual innocence of the person being interrogated and that people under interrogation would feel either anxiety for true detection or anxiety for false detection. The anxiety also entails both cognitive and affective components–attempts to hide or regulate anxiety reflected in the facial expressions or physiological responses.

Current results showed that there were distinct brain networks that were involved in deceptive acts and their post-hoc responses (guilt condition) and the anxious responses for interrogation (after engaging in either deception or truth-telling, within the interrogation condition); brain responses in the guilt condition mainly recruited regions in the central-executive network and the basal ganglia-thalamic networks, while responses under interrogation mainly recruited regions in the salience network, theory of mind networks, and sensory-motor networks, among multiple brain networks classified by Menon and Uddin [23]. Regardless of whether the person has engaged in deception or not, interrogation itself gave rise to anxiety, which was reflected in the brain activation as well as post-hoc self-reports. By analyzing interaction effects, we further detailed neural correlates of two different types of anxieties- one caused by the actual deception (true positive) and the other caused by the fear of being falsely accused (false positive).

Guilt conditions, relative to innocence conditions, yielded higher involvement of brain areas previously reported to be associated with deception [4, 9–11, 18–20], with a strong right-hemispheric dominance. The deception-related brain regions primarily corresponded to the central-executive network and thalamo-basal ganglia network. The prefrontal regions have been established as centers for executive control such as response inhibition and error-monitoring, and are known to play a role in deceptive behavior [9, 18, 24]. In particular, the superior frontal gyrus is reported to be involved in lying about affective stimuli [25], and the right inferior frontal gyrus in mediating successful lying [26]. The supramarginal gyrus has been implicated in general deception processes related to action execution, simulation, observation, and working memory [5, 27–29]. The left supramarginal gyrus turned out to be a region showing fairly high decoding accuracy for holding true-thoughts even during telling a lie on a cue, suggesting that the left supramarginal gyrus is responsible for encoding true thoughts [12]. The caudate and thalamus have been associated with suppression of prepotent (truthful) responses [19]. Recruitment of these areas can be understood in the context of cognitive and affective processes necessary for lying and its consequent behaviors, e.g., suppression of honest responses, thinking about what the interrogator might be thinking about, prospection about lying during the act of deception, and retrospection on lying afterwards.

We assigned the onset time at the beginning of interrogation (or no-interrogation phase) to explore the effects of interrogation, and denoted the state after the onset time (following the deceptive act) as a guilt state rather than a deception state. Therefore, brain regions detected in the guilt-innocence contrast may reflect post-hoc cognitive and affective processes that differentially unfold after lying or truth-telling, regardless of interrogation. We, however, note a technical issue that brain signals generated during a lie and right after a lie could not be fully separated from each other in this experiment. This is due to the nature of the blood oxygenation level dependent (BOLD) signal in fMRI. Although a deceptive act occurred two seconds before the onset time, BOLD signals of deception processes would sustain, albeit being relatively weakened (due to the very slow pace of hemodynamic response, which peaks around 6 secs after an actual neurodynamic event). In addition to the acting phase, there is also the waiting period for the decision of whether there would be interrogation or not. The common neural processes that unfold regardless of whether a participant lied or not during this waiting period, such as anxiety for the decision about the imminent interrogation, we assumed, were cancelled in the ANOVA analysis. Meanwhile, the differential processes during the waiting period after lie or truth-telling would be embedded in the main effects of the guilt-innocence contrast. Owing to the scope of the current design, details of neural processes occurring in the acting phase and post hoc phase, common to both Interrogation and No-interrogation, remains to be explored further by future studies.

As a main research goal of the current study, interrogation conditions elicited higher activation in the salience network (anterior insula and cingulate cortex) and a network for theory of mind (superior temporal sulcus, middle temporal gyrus and supramarginal gyrus) compared to no-interrogation conditions, regardless of lying or truth-telling.

The anterior insula, as a center for the salience network, is known to be responsible for integrating and modulating sensory information coming from the body and its activity has been correlated with processing unpleasant emotions [4, 30–32]. It is also involved in emotional awareness[33], cognition-emotion integration [34], producing cardiovascular effects (both sympathetic and parasympathetic) [35], and interoception [36]. All these functions of the anterior insula appear to be at play as participants sense their own emotional arousal (anxiety under interrogation) as well as the physiological state of the body (interoception), and deal with competing inputs from both cognitive and emotional aspects of the unpleasant interrogatory experience.

Increased activation in the dorsal anterior cingulate cortex, as a part of the salience network, can also be understood along the same lines. The anterior cingulate cortex is known for its involvement in a variety of functions, including error detection [37], conflict monitoring and response selection [38], and regulation of emotions [39–41], all of which can readily be understood as processes relevant in an interrogative context. Specifically, the dorsal anterior cingulate cortex is reported to be involved in vigilance due to anticipatory anxiety [42]. Activation in these anxiety-related areas during the interrogation conditions is also confirmed by the post-hoc questionnaires as participants reported having been conscious of the interrogation setting and the corresponding anxiety arising from that awareness.

The superior temporal area and supramarginal gyrus, parts of the temporal parietal junction [43], had higher activations during the interrogation. Those regions have previously been reported to be implicated in social cognition [44, 45], intention detection, and theory of mind [46, 47]. The middle temporal gyrus has been implicated in social anxiety induced by guided mental imagery in patients with generalized social anxiety disorder [48]. Those areas belonging to the theory of mind network can be thought of as components of the mentalization process about the interrogator.

The involvement of sensory-motor networks (facial area in the precentral gyrus and premotor area, the anterior supplementary motor area, and the occipital areas) during interrogation was not expected. However, recruitment of the sensory-motor network for the interrogation may be explained by the processes the participants went through in interpreting sensory information and motor control during the interrogation, such as evaluating their own facial expressions and biophysical signals as well as adjusting them, to successfully deceive the interrogator. This was also confirmed through the post-hoc self-reports.

As expected, the no-interrogation condition, in contrast, produced activations in the default mode network, including bilateral posterior cingulate, bilateral precuneus, and the right angular gyrus as part of the inferior parietal lobule, which has been previously known to be active during cessation of attention-demanding tasks [15–17]. These areas, as part of the default mode network, appear to be associated with the neural mechanisms underlying a relief response in the context of a cancelled interrogation session (no-interrogation conditions).

Interestingly, the activation pattern for interrogation response was highly bilateral in contrast to the right-hemispheric dominance we observed in the Guilt > Innocence contrast.

Although the main effect for brain responses under interrogation regardless of truthfulness was strong and widely distributed, we still found some differential neural responses between the contexts of true positive anxiety and its concurrent behavior (fear of being caught) compared to false positive anxiety and its entailing behavior (fear of being falsely accused of an act he/she did not commit). This was reflected in the interaction effects between the type of acts (truth vs. lie) and the interrogation administration (presence vs. absence of interrogation). The detected regions were the bilateral thalamus and caudate, left cerebellum, bilateral ventral anterior cingulate cortex and ventromedial prefrontal cortex. All those brain regions were suppressed for conditions except for interrogation after deception, which showed increased activation or no significant suppression (Fig 3).

The caudate and thalamic nuclei are regions known to be recruited during response inhibition and motor control [19, 20, 49, 50]. The cerebellum has previously been reported to be associated with the act of deception and anxiety [9, 18]. The ventral anterior cingulate cortex, which is known to play emotional regulation [51], is often considered together (in terms of coordinates or its function) with the ventromedial prefrontal cortex in previous studies [52–54]. The ventromedial prefrontal cortex is recruited for the regulation of parasympathetic activity in the fear and risk processing and is activated during suppression of affective responses to a negative emotional signal [52]. This is consistent with a lesion study that the focal damage in the ventromedial prefrontal cortex induces potentiated amygdala responses to aversive images [54]. Meanwhile, patients with damage at the ventromedial prefrontal cortex, show inability to generate guilty feelings in situations of moral judgement [53], supporting the role of the ventromedial prefrontal cortex in the normal generation of social emotions. The current finding for interactions at those regions is understandable because one would expect to observe more tension (negative affect as it relates to interrogation) and disguise tactics (suppression of honest responses and manipulation of facial and posture cues) in guilt conditions than in innocence conditions, although both cases involve the same interrogative context.

In summary, the anxious response to interrogation recruits specific brain circuits, segregated from the brain regions responding to guilt, which should be considered in lie detection systems involving interrogation. This argument is apparent and is not new. In practice, when considering biophysical monitoring data in lie detection, interrogators have been advised to differentiate between these two different factors affecting physiological signals. In line with this, we propose that fMRI data concerning the distinctly distributed neural patterns of different condition types could be utilized to identify these two factors.

As a limitation of the current study, we did not obtain anxiety scores for performing each trial during the fMRI experiment in order to make the current experimental setting realistic, by not giving a hint to participants that the interrogation judgment was based on the self-report on the anxiety level. If we had acquired biophysical measurements linked to the self-reported anxiety level, it would have been helpful in the interpretation of current results. Furthermore, the relatively small sample size (N = 19) analyzed in the current study does weaken the strength of our conclusions. Further studies with a sufficient sample numbers could help increase the reliability of our findings.

The present study attempted to test and provide neurobiological evidence for the common knowledge that interrogation causes anxiety and anxiety-related responses in both the deceptive and the innocent. Under interrogation, regardless of the participant’s innocence, there was higher activation in the salience network, the theory-of-mind network, and the sensory-motor network, compared to that in no-interrogation conditions. We also found interaction effects within the interrogation conditions, signifying the differential neural responses associated with the two types of anxiety- true positive and false positive—as hypothesized. Future studies can try to measure the accuracy with which deceptive and truthful trials can be discriminated by using fMRI data, while also making further efforts to delineate the distinct activation patterns of deceptive and innocent responses under interrogation. One can then hope that these fMRI data will help bring more precision to court judgments.

## Supporting information

S1 Data (DOCX)
