# Supplementary material for: Neural correlates of anxiety under interrogation in guilt or innocence contexts
Source: PLoS One. 2020 Apr 9;15(4):e0230837. doi: 10.1371/journal.pone.0230837 (PMC7145196; doi:10.1371/journal.pone.0230837)
Supplement: S1 Data — (DOCX) [file pone.0230837.s001.docx]

**<Post hoc Questionnaire>**- translated from Korean

1. How was your overall experience of the experiment? Please express your level of concentration on the scale of 1 (Low concentration) to 5 (High concentration).

A-1. If the concentration was low, why is that?

1. If you have any feelings or any comments about the National Forensic Service's investigation, please write them down.
2. What do you think was the accuracy of the interrogator’s judgment in each session? (0-100%)

-First session

-Second session

1. Please write down what you thought about during the scan.

1. There were four possible situations in the game that you went through. Please indicate your anxiety level and describe what you thought about during each situation.

| 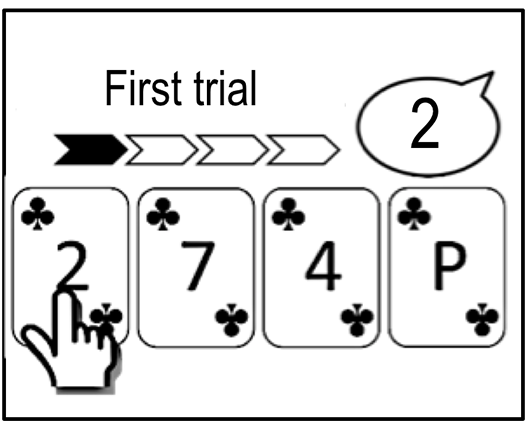  Choice of  compatible card | 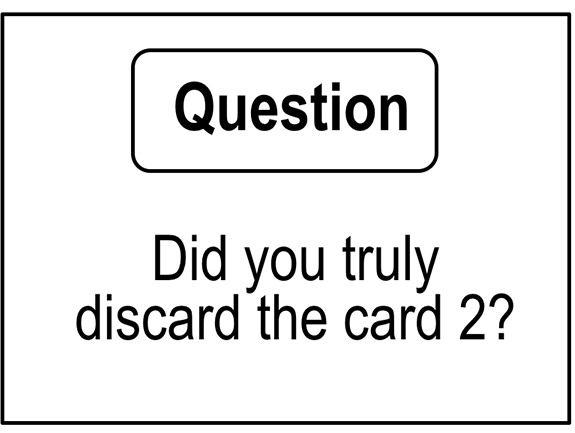  Interrogation | 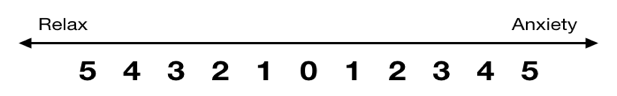 |
| --- | --- | --- |
|  |  |  |
|  | 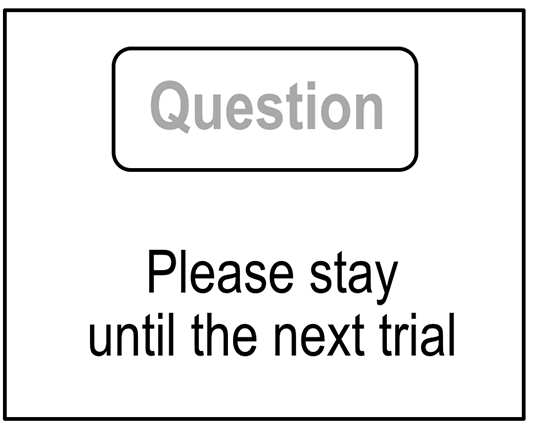  No interrogation | 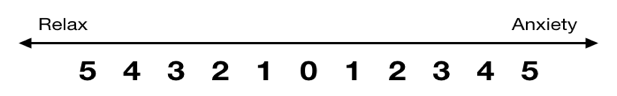 |
|  |  |  |
| 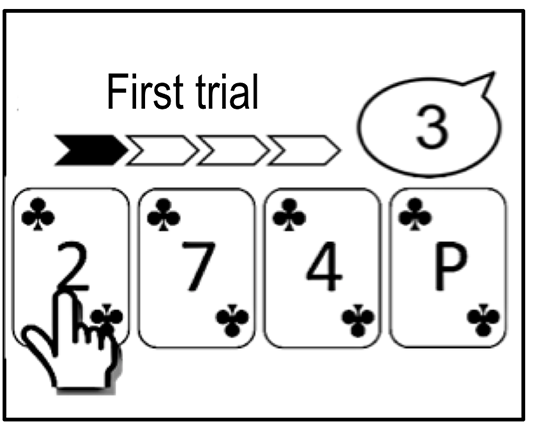  Choice of  incompatible card | 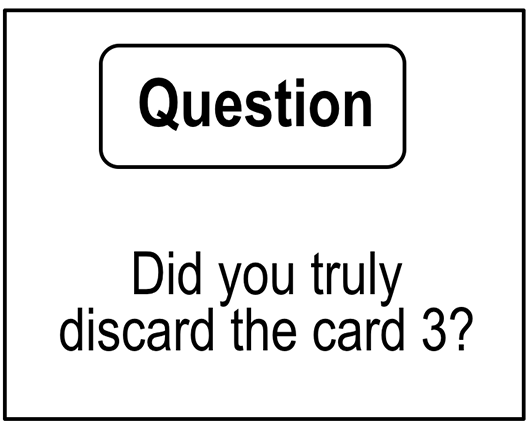  Interrogation | 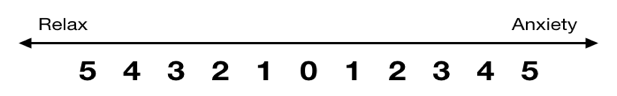 |
|  |  |  |
|  | 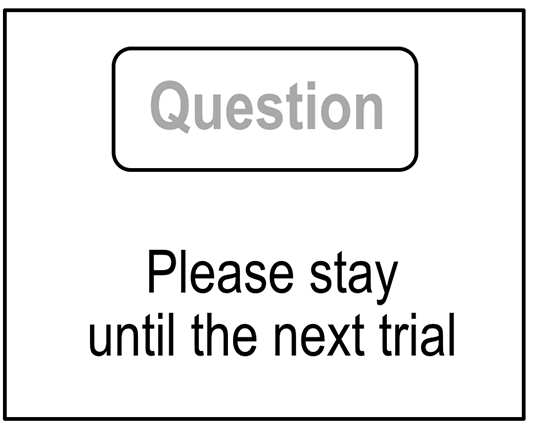  No interrogation | 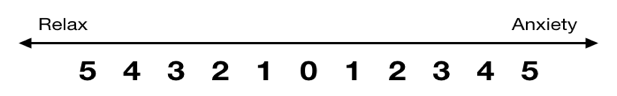 |
|  |  |  |
